# Reflective practice improves Basic Life Support training outcomes: A randomized controlled study

**DOI:** 10.1371/journal.pone.0287908

**Published:** 2023-06-29

**Authors:** Marie S. Thommes, Michelle Schmidt, Sophie I. Lambert, Michael T. Schauwinhold, Martin Klasen, Saša Sopka

**Affiliations:** 1 AIXTRA – Competence Center for Training and Patient Safety, Medical Faculty, RWTH Aachen University, Aachen, Germany; 2 Department of Anaesthesiology, University Hospital Aachen, Medical Faculty, RWTH Aachen University, Aachen, Germany; University Medical Centre Ljubljana (UMCL) / Faculty of Medicine, University Ljubljana (FM,UL), SLOVENIA

## Abstract

**Introduction:**

Practical skills training is an essential part of medical education. An important example is the training of Basic Life Support (BLS) skills, which are key to improve patient outcomes in life-threatening situations. However, despite practical training, BLS performance is often sub-optimal even among healthcare professionals and medical students. Finding more effective training methods is therefore of high importance. A promising method to enhance learning outcomes is reflective practice. The goal of the present study was to evaluate whether a short reflective practice intervention following standard BLS training (Peyton’s 4-step approach) improves BLS training outcomes, reflected in higher BLS performance and higher self-confidence to perform BLS.

**Method:**

287 first-year medical students were randomly assigned to one of two BLS training conditions: 1) standard BLS training (ST), 2) ST followed by a 15-minute reflective practice exercise. Outcome parameters included objective BLS performance data assessed by a resuscitation manikin, and students’ self-reported confidence in their BLS skills. Outcomes were assessed directly after the training (T0) and re-assessed one week later (T1). A two-way mixed model analysis of variance (ANOVA) was conducted to examine the effect of the intervention on BLS performance and self-reported confidence. Significance was determined by two-sided 95% confidence intervals.

**Results:**

The intervention group performed significantly more effective compressions at T1 and began significantly faster with performing their first chest compression at T0 and T1, in comparison to the control group. No significant differences between study groups regarding their self-reported confidence to perform BLS were observed.

**Conclusion:**

This research shows that standard BLS training accompanied with a simple, cost-effective reflective practice exercise can improve learners’ BLS skill acquisition and retention. This shows that reflective practice has the potential to enhance practical skills training in medicine; yet, more empirical studies are needed to examine its broader applicability.

## Introduction

Practical skills training is an essential part of medical education. An important example is the training of Basic Life Support (BLS) skills, which are key to improve patient outcomes in life-threatening situations [1, 2]. If applied effectively, BLS decreases mortality and morbidity following sudden cardiac arrest (SCA)–one of the main causes of death [3]. Furthermore, the time to apply BLS should be as short as possible to improve patient outcomes [4–6]. Despite the importance of timely and effective BLS provision, BLS performance is often sub-optimal and quickly declines after initial training, even among healthcare professionals and medical students [7]. Accordingly, improving educational methods for BLS training to enhance BLS skill acquisition and retention is essential and has been highlighted by official resuscitation guidelines (e.g., European Resuscitation Council; ERC) and medical educational committees (e.g., Association of American Medical Colleges; AAMC) [8, 9]. However, supporting future healthcare providers in acquiring and retaining basic practical skills is often time consuming and can be costly, as it typically requires continuous guidance by an expert instructor. Improving training to enhance learners’ skill acquisition and retention while keeping resource investment minimal therefore remains a key challenge in basic medical education. This challenge becomes particularly apparent during pandemic times when human resources are scarce.

Reflective practice has been put forward by medical curricula as a promising tool to enhance student learning outcomes through deliberate and active processing of acquired knowledge and feedback [10–13]. However, evidence is currently lacking regarding how reflective practice may support student learning in the context of basic medical competencies training, as well as how it can be effectively implemented in such settings [14]. In this study, we aim to address this gap by combining standard BLS training with a reflective practice exercise to improve BLS training outcomes. Thereby, we built on research from psychological science and medical education that offer theoretical and practical guidance for using reflective practice as an instrument to enhance students’ competencies acquisition and retention.

Reflective practice is broadly defined as “a metacognitive process that creates a greater understanding of both the self and the situation so that future actions can be informed by this understanding” [15]. According to established theoretical models in educational literature (e.g., Kolb’s reflective model, Gibb’s reflective cycle), this process typically follows several cyclical steps including 1) understanding the experience by evaluating what worked and what didn’t work in a concrete performance situation, 2) concluding which changes are required to improve an outcome in the future, and 3) formulating an action plan on how to concretely act in a similar future situation [16, 17]. Previous research provided support for the argument that reflective practice enables learners in better identifying gaps between their current and optimal performance and in preparing for, and successfully dealing with, similar future events [10, 18, 19]. Additionally, the elaborate information processing through reflective practice does not only assist students with internalizing acquired knowledge but also increases their ability to recall and retain learning outcomes over longer periods of time [20]. Additionally, preliminary findings suggest that while reflective practice may increase uncertainty in some learners, it generally promotes individuals’ confidence in their skills and their desire for self-improvement [21].

Following this research, the aim of this study was to examine reflective practice as a tool to improve the learning outcome of standard BLS training (Peyton’s 4 step approach). For this purpose, we implemented a standardized reflective practice exercise to induce structured reflection on learners’ previous action and examined whether standard BLS training combined with structured reflective practice (1) increases students’ BLS performance (i.e. amount of effective compressions), (2) reduces their time to start performing BLS (i.e. time to first chest compression), and (3) increases students’ confidence in their BLS skills.

## Materials and methods

### Ethics

Ethical approval (Ethical Committee 407/21) was granted according to the ethical principles of the World Medical Association’s Declaration of Helsinki [22] on 26.10.2021 by the Ethical Committee of the RWTH Aachen University Hospital, Pauwelsstraße 30, 52074 Aachen, Germany (Chairperson Prof. Dr. med. G. Schmalzing).

### Participants

287 undergraduate first year medical students participated in the study as part of a mandatory introductory course on emergency medicine during the first three weeks of their studies. Data collection took place in October 2021.

### Study design

Prior to the BLS training, students were informed about the study and were assured that their participation was voluntary and would not affect their course grade. To be eligible for study participation, students had to provide written informed consent. Subsequently, students received a short pre-questionnaire in which they were asked to report their previous medical education and prior BLS training experience. Participants were then randomly assigned to either a control group or an intervention group. Both study groups received a BLS training according to Peyton’s 4 step teaching approach, which is commonly used to teach procedural skills in medical education [23]. In Step 1, a trained instructor demonstrated BLS to the medical students. In Step 2, the instructor repeated the demonstration while providing a step-by-step explanation of the different relevant actions. In Step 3, the instructor performed BLS based on trainee instructions. In Step 4, each participant performed BLS on a *Resusci Anne*^*TM*^ manikin (Laerdal, Stavanger, Norway) in smaller groups (12 persons) and received feedback on their performance from a trained instructor. This procedure is in line with Peyton’s 4 step approach and has been investigated in previous studies [24–26].

The BLS training lasted 90 minutes in total including 30 minutes for Steps 1–3 and 60 minutes for Step 4. In the last step, 5 minutes of individual training time was available per participants. First, participants were asked to provide an initial assessment followed by approximately 45 seconds of chest compressions and ventilations with simultaneous feedback. In a second run, participants applied the initial assessment followed by 120 seconds of chest compressions and ventilations with final feedback. Based on this process, participants were given the chance to implement feedback from the first round. With 30 chest compressions according to the guidelines, one sequence of compressions would last from 15 (for 120 compressions/min) to 18 seconds (for 100 compressions/min). Including ventilations, we conservatively estimate that a compression-ventilation sequence lasts no longer than 30 seconds. Therefore, participants were able to complete at least 5 complete compression-ventilation sequences in the 165 seconds of practical training time. Moreover, every participant had the chance to actively watch the performance of the group members and listen to the feedback provided to them as well. The study groups only differed in the training method provided after the final step of the training:

#### Control group

Following the standard BLS training (ST) explained above, students started the first assessment (T0).

#### Intervention group

**Fig 1:**
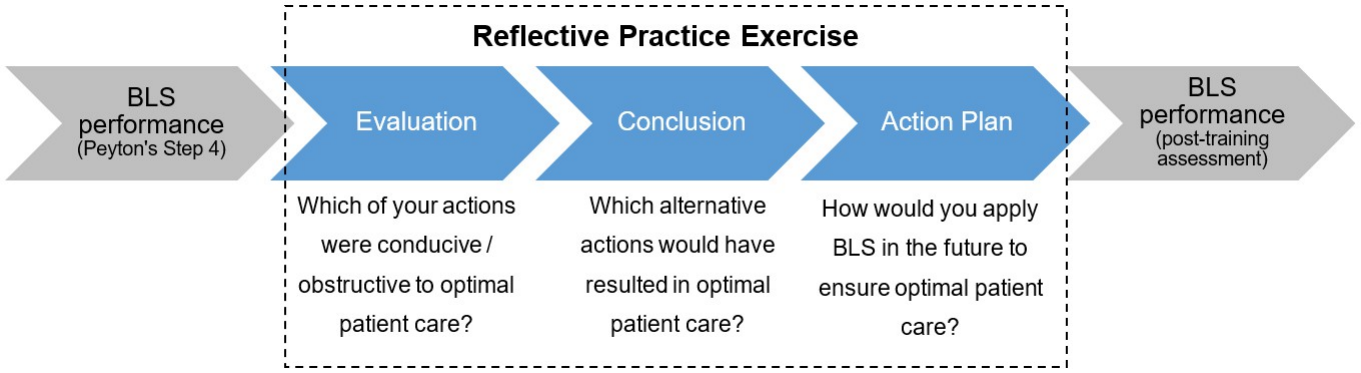
Reflective practice exercise.

The intervention group followed the same procedure as the control group; however, participants additionally received a guided reflective practice exercise after the ST and before the first assessment. The exercise consisted of three questions based on self-reflexivity principles that are consistently included in theoretical reflection models [16, 27]. The questions were presented to participants in written form [28]. The exercise lasted for 15 minutes, in which participants were asked to reflect on their BLS performance during the training (Step 4) and to provide specific information on (1) personal actions during the training that were conducive / obstructive for optimal patient care, (2) alternative actions that would have resulted in optimal patient care, and (3) a concrete action plan on how to apply BLS in the future to ensure optimal patient care. A similar intervention has previously been used in a team training setting (29). Participants provided their answers anonymously on the written form. Fig 1 depicts the reflective practice exercise and corresponding self-reflective principles.

**Fig 2:**
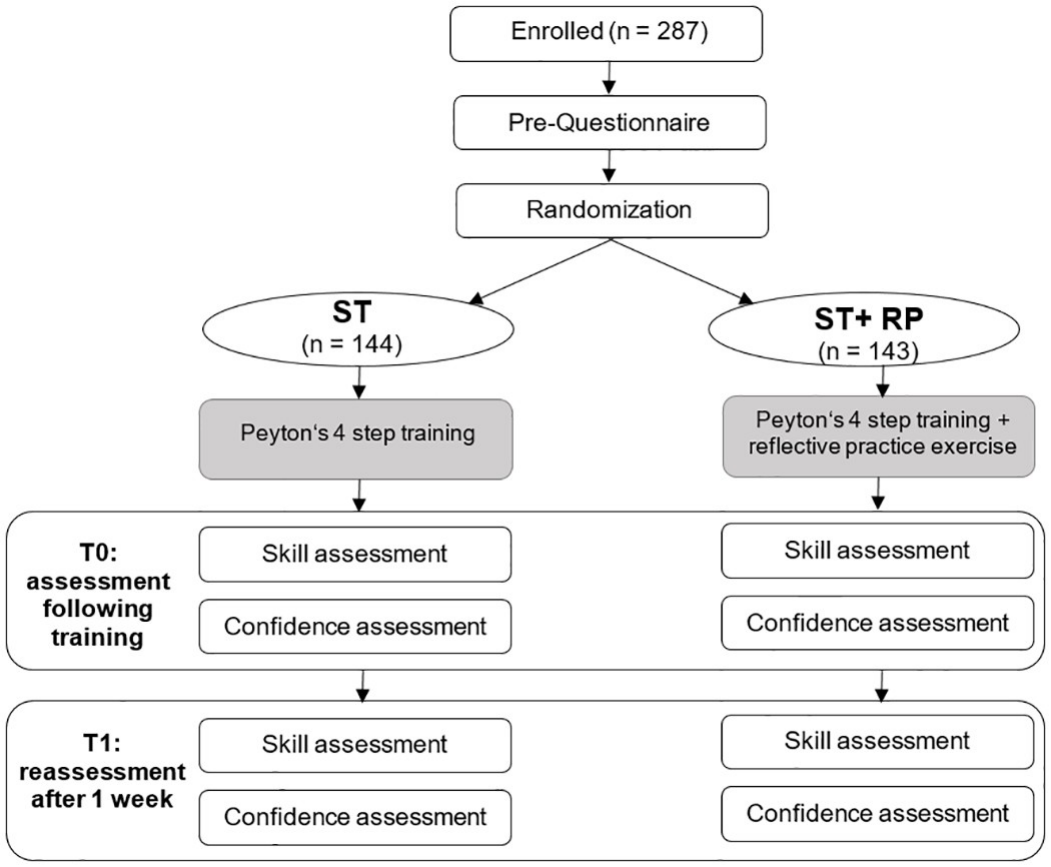
Flow chart.

Upon completion of the BLS training, participants completed a short online questionnaire and were asked to provide BLS to a *Resusci Anne*^*TM*^ manikin (Laerdal, Stavanger, Norway) that was positioned on the floor. Participants were instructed to imagine that they had just witnessed a person collapsing and to follow the trained BLS protocol. The scenario started as soon as the participant indicated they had understood the instructions and finished 120 seconds after the participant performed the first chest compression on the *Resusci Anne*^*TM*^ manikin. A flow chart of the study is depicted in Fig 2.

### Research question

The research question was designed according to the PICO framework [29] including the following elements:

**(P) Population**: Undergraduate first-year medical students attending a BLS training
**(I) Intervention**: Participants completing a reflective practice exercise after BLS training
**(C) Comparison**: Participants completing no reflective practice exercise after BLS training
**(O) Outcome**: There is an effect on the number of effective compressions when participants are given the possibility to participate in a reflective practice exercise after BLS training.

The above defined PICO elements lead to the formulation of the research question as follows: *In undergraduate first-year medical students attending a BLS training (P)*, *what is the effect of a reflective practice exercise after training (I) compared with no reflective practice exercise after training (C) on the number of effective compressions (O)*?

### Measures

The post-interventional assessments took place directly following the training (T0) and one week after the training (T1), using the same standardized clinical scenario described above.

#### Skill assessment

*BLS performance* was assessed by the amount of effective compressions, defined as the number of compressions within the correct depth range of 50–60 mm, correct decompression and correct hand position, as proposed by the American Heart Association (AHA) guidelines [30]. This outcome parameter indicates the extent of efficient hemodynamic support and thereby reflects the effective provision of CPR and was recorded by the manikin’s Laerdal PC Skill Reporting Software. The time interval of the recording started with the first chest compression provided by the participants and ended 120 seconds later.

*Time to start BLS* included the time that had passed between the start of the clinical scenario and the first chest compression provided by the participant. The time was recorded by a certified BLS instructor who supervised the data recording and who was blind to the study conditions.

#### Confidence assessment

To assess students’ self-reported confidence in their BLS skills, participants were asked to rate their confidence to provide BLS. Answers were provided on a 6-point Likert Scale ranging from 1 (not at all confident) to 6 (very confident).

### Sample size planning

Sample size planning was performed with the G*Power software version 3.1.9.7 [31]. The calculation was conducted using an analysis of variance (ANOVA; repeated measures, between effects) with two groups and two measurement times (T0 and T1). Assuming a small effect of f = 0.20, an alpha error level of 0.05 and a power (1-β) of 95%, this resulted in a total sample of *N* = 246. The sample size was met for all outcome parameters.

### Randomization

Prior to the start of the study, students were assigned to groups of 12 by an administrative university employee who was blind to the study. Next, student groups were randomly assigned to intervention or control group, using a web-based randomizer [32].

### Statistical analysis

Data were analyzed with IBM SPSS Statistics Version 25 (IBM Corp., Armonk, NY, USA). A two-way mixed model analysis of variance (ANOVA) with time as the within subject factor and study group as the between subject factor were conducted to examine the effect of the intervention on BLS performance, time to start first compression, and students’ self-reported confidence, respectively. Significance was determined by two-sided 95% confidence intervals.

Raw data of the study underlying all analyses reported in this paper can be found under S1 Raw data.

## Results

### Sample characteristics

The total number of students participating in this was study was 287. 64,5% (*n* = 160) of the sample was female and the average age was 20.34 years (*SD* = 3.00). Sample sizes may vary for the different analyses due to single missing data points.

### Randomization check

To test whether randomization was successful, a chi-square test of independence was conducted comparing the frequency of prior BLS training participation, as well as prior medical education in the two study groups (intervention/control). Results indicate no significant differences in frequencies regarding prior BLS training participation (*X*^2^ (6, *N* = 258) = 3.39, *p* = .76), nor regarding previous medical education (*X*^2^ (5, *N* = 258) = 8.71, *p* = .12) between the two study groups. These results suggest that randomization was successful.

### Descriptives

Table 1 reports the descriptives of students’ BLS performance parameters, time to start first chest compression and self-reported confidence at T0 and Table 2 for T1 for the intervention group (ST + reflective practice) and control group (ST only).

**Table 1:** Descriptive data on BLS performance and self-reported confidence to perform BLS in intervention and control group at T0.

T0 (immediately after training)				
	Control group		Intervention group	
	*M*	*SD*	*M*	*SD*
Amount of effective compressions (absolute number)	63.86	61.03	68.88	59.75
Time to start first compression (sec)	28.64	8.57	25.32	6.82
Self-reported confidence to perform BLS (6-point Likert scale)	5.17	0.80	5.22	0.85

**Table 2:** Descriptive data on BLS performance and self-reported confidence to perform BLS in intervention and control group as well as intergroup comparisons from one-way ANOVA at T1.

T1 (1 week after training)					
	Control group		Intervention group		Group differences
	*M*	*SD*	*M*	*SD*	*p*
Amount of effective compressions (absolute number)	57.24	54.80	71.12	56.04	.04
Time to start first compression (sec)	28.65	9.05	24.57	7.60	**< .001**
Self-reported confidence to perform BLS (6-point Likert scale)	5.33	0.66	5.24	0.67	.17

### Analysis

Results of the statistical analysis (two-way mixed model ANOVA) are reported in Table 3.

**Table 3:** Two-way mixed model ANOVA results.

Source	Effective compressions			Time to start first compression			Confidence to perform BLS		
	*df*	*F*	*p*	*df*	*F*	*p*	*df*	*F*	*p*
Between-subjects effects									
Study group	1	2.55	.11	1	18.86	**.001**	1	0.16	.69
Within-subjects effects									
Time	1	0.17	.68	1	0.09	.77	1	3.88	.05
Interaction									
Time*Study group	1	0.66	.42	1	0.61	.48	1	0.23	.63

*Note*. Time = within-subject factor; Study group = between-subject factor; *N* = 258 included in the analysis for effective compression and time to start first compression

#### Primary outcome parameter: BLS performance

*BLS performance*. Results of a two-way mixed model ANOVA showed no significant effect for the amount of effective compressions, *F* (1, 256) = 2.55, p = 0.11. However, an additional analysis using one-way ANOVA revealed a main effect of the intervention on the amount of effective compressions in the expected direction at T1 (one week after training). That is, the intervention group demonstrated a significantly higher amount of effective compressions defined as number of compressions within the correct depth range of 50–60 mm, correct decompression and correct hand position in comparison to the control group after one week of BLS training, *F* (1, 264) = 4.17, *p* = 0.04.

#### Secondary outcome parameters

*Time to start first compression*. Results of a two-way repeated measures ANOVA further revealed a main effect of the intervention on the time to start with BLS in comparison to the control group. The intervention group took significantly less time to start with the first compression in comparison to the control group overall, *F* (1, 256) = 18.86, *p* < 0.01.

*Self-reported confidence in BLS skills*. No significant differences between intervention group and control group regarding their self-reported confidence to perform BLS were observed using two-way repeated measures ANOVA, *F* (1, 186) = 0.16, *p* = 0.69. Additional analyses using one-way ANOVA further revealed no significant differences between the two study groups at T0 (*F* (1, 238) = 0.27, *p* = 0.61), nor at T1 (*F* (1, 212) = 0.97, *p* = 0.33).

## Discussion

This study investigated whether a short reflective practice exercise increases the effectiveness of an established BLS training. Results confirmed that combining a structured reflective practice exercise with a standard BLS training improves students’ timely and effective provision of BLS over the period of one week. No significant differences were observed between the two study groups regarding their self-rated confidence in their BLS skills.

These findings highlight the great potential of reflective practice to improve traditional BLS education by increasing students’ ability to internalize and retain learning outcomes. The functionality of reflective processing is increasingly discussed in medical education research and has long been highlighted in the domain of cognitive psychology by the levels of processing theory of memory [20]. This theory proposes that memory is a function of the depth with which learning material is processed by the learner, regardless of the amount of repetition of the material. Accordingly, empirical studies provide evidence for a positive relation between meaningful processing of information and learning outcomes [33]. In line with these findings, our observations confirm the potential of reflective, deep-level processing for improving learning outcomes in the context of BLS training. Particularly, our findings suggest that reflective practice improves the recall of learned information, as reflected by the reduced starting time to perform BLS in the intervention group at both assessment points (i.e., immediately after initial training and one week later). Further, the finding that students engaging in reflective practice performed significantly more effective compressions one week after initial training highlight the potential of reflective practice for improving the retention of practical skills.

Another clear strength of reflective practice is that costs and infrastructural requirements are minimal. The reflective practice exercise as applied in this study solely required a short written instruction. The great advantage of this method is that it can be easily implemented in any setting. The reflective practice exercise was based on standardized questions and did not require any guidance by an expert instructor. This is particularly useful to increase training effectiveness in BLS training settings in which professional instructors are lacking (e.g., in schools or companies).

Additionally, this method can make acquired knowledge more easily accessible and refreshable to learners outside the training environment. Previous studies showed that without any training refreshment, BLS knowledge and practical skills significantly deteriorate within three to twelve months [34]. As demonstrated in this study, reflective practice has the potential to reduce such performance loss, potentially even for longer periods of time. It therefore seems promising to augment self-directed refresher training without instructors using the reflective practice method. Taking into account an objectifiable evaluation of the training, e.g. by means of a video feedback method with peer learners [24], this would possibly lead to equivalent learning outcomes as instructor-based refresher training and enable significant resource efficiency. Further research is needed to investigate this topic more in-depth.

Furthermore, our findings show that reflective practice did not influence students’ self-rated confidence to perform CPR over the period of one week. This observation does not confirm previous preliminary findings suggesting that reflective practice can increase uncertainty or self-doubt in individuals [21]. These inconsistencies may be explained by the structure of the reflective practice exercise as applied in this study. The reflective practice exercise consisted of standardized questions to assist students in engaging in a *structured* reflection process (i.e., evaluation, conclusion, action plan). Particularly the formulation of a concrete action plan prepares students for applying BLS in the future, thereby counteracting uncertainty that may have initially increased through the critical evaluation of personal actions. It is therefore rather likely that reflective practice, if more frequently applied, increases confidence in practical skills over longer time intervals; for example, by increasing awareness of personal strengths or by mentally rehearsing specific practical steps that require improvement [14].

Finally, while this study focuses on the use of reflective practice in the context of BLS, it is likely reflective practice may also improve basic medical education in other areas in which basic practical skills have to be acquired, internalized and effectively applied (e.g., wound suturing, knots). Combining reflective practice with traditional teaching therefore provides a promising didactic approach that has the potential to improve basic practical skills training in healthcare in a cost-effective way.

### Limitations

Like any research, this study has some limitations that should be considered when drawing conclusions. First, our assessment took place over the period of one week. At this point, this research does not allow any conclusion regarding the influence of reflective practice on BLS training effectiveness over longer periods of time. Considering previous evidence that BLS performance significantly drops within a three to six months period after BLS training [34], and given the potential of reflective practice to mitigate such negative effects, it seems highly interesting and important to examine long-term effects of reflective practice in a BLS context.

Additionally, our sample consisted of a medical student population with characteristic features (e.g., young age, academic background, medical interest). The generalizability of study findings to other populations is therefore subject to future research. For example, it is possible that learners’ educational background or personal interest in developing medical skills influence the ability or motivation to engage in reflective practice in a BLS training context. The use of reflective practice to increase BLS training effectiveness may therefore differ depending on the trainee population.

To address the widespread lack of personnel and time resources, we aimed to implement a time and resource efficient method which still has the ability to improve learning outcomes. However, future research should investigate this method further and compare the effectiveness of the reflective practice to other interventions that aim for improving performance outcomes in BLS training (e.g. training time). Lastly, the pure hands-on training time for each participant was rather short due to limited time resources. Wherever possible, future research should extend the time for participants to apply their resuscitation skills.

### Conclusion

Timely and effective BLS provision is crucial; it can increase survival rates and improve patient outcomes after SCA. Enhancing the effectiveness of BLS education is therefore of high importance. This study illustrates that standard BLS training combined with a short, structured reflective practice exercise has the potential to improve students’ BLS acquisition and retention. These findings highlight reflective practice as a promising tool that has the potential to improve the internalization of BLS skills, thereby improving the effectiveness of traditional BLS education.

## Supporting information

S1 Raw data Raw data underlying the analyses. (XLSX)
